# Improving radiotherapy in cancer treatment: Promises and challenges

**DOI:** 10.18632/oncotarget.18409

**Published:** 2017-06-08

**Authors:** Helen H.W. Chen, Macus Tien Kuo

**Affiliations:** ^1^ Division of Clinical Radiation Oncology, Department of Radiation Oncology, National Cheng Kung University Hospital, Department of Radiology, College of Medicine, National Cheng Kung University, Tainan, Taiwan; ^2^ Department of Translational Molecular Pathology, The University of Texas MD Anderson Cancer Center, Houston, Texas, USA

**Keywords:** radiotherapy, DNA damage response, hypoxia, cancer genomics, immune check points

## Abstract

Effective radiotherapy for cancer has relied on the promise of maximally eradicating tumor cells while minimally killing normal cells. Technological advancement has provided state-of-the-art instrumentation that enables delivery of radiotherapy with great precision to tumor lesions with substantial reduced injury to normal tissues. Moreover, better understanding of radiobiology, particularly the mechanisms of radiation sensitivity and resistance in tumor lesions and toxicity in normal tissues, has improved the treatment efficacy of radiotherapy. Previous mechanism-based studies have identified many cellular targets that can affect radiation sensitivity, notably reactive oxygen species, DNA-damaging response signals, and tumor microenvironments. Several radiation sensitizers and protectors have been developed and clinically evaluated; however, many of these results are inconclusive, indicating that improvement remains needed. In this era of personalized medicine in which patients’ genetic variations, transcriptome and proteomics, tumor metabolism and microenvironment, and tumor immunity are available. These new developments have provided opportunity for new target discovery. Several radiotherapy sensitivity-associated “gene signatures” have been reported although clinical validations are needed. Recently, several immune modifiers have been shown to associate with improved radiotherapy in preclinical models and in early clinical trials. Combination of radiotherapy and immunocheckpoint blockade has shown promising results especially in targeting metastatic tumors through abscopal response. In this article, we succinctly review recent advancements in the areas of mechanism-driven targets and exploitation of new targets from current radio-oncogenomic and radiation-immunotherapeutic approaches that bear clinical implications for improving the treatment efficacy of radiotherapy.

## INTRODUCTION

Radiotherapy is used in at least two-thirds of cancer treatment regimens in Western countries, and remains an important curative treatment modality for uncomplicated locoregional tumors. Over the past few decades, substantial technological advancement in 3D conformal radiation treatments, such as stereotactic (body) radiotherapy (SBRT), intensity-modulated radiation therapy (IMRT) and improved imaging systems (i.e., image-guided radiation therapy, IGRT), have enabled precise delivery of matching radiation doses to the exact dimensions of tumor while minimizing radiation exposure of surrounding normal tissue (see recent review [1]). These state-of-the-art technological advancements, together with a better understanding of tumor biology at the molecular, cellular, and physiological and immunological levels, have improved the treatment efficacy of radiotherapy. For example, the overall survival rates of cancer radiation therapy have improved from about 30% two decades ago to about 80% nowadays in some malignancies such as head and neck cancers [1, 2]. However, many tumor types remain insensitive to radiotherapy owing to intrinsic resistance or recur shortly after the treatment because of acquired resistance. Cancer stem cells are considered to be the primary source of radiation- and chemo-resistace, and tumor heterogeneity plays important role in acquired radiation resistance, indicating that radiotherapy still needs to improve [3].

Traditionally, cancer radiotherapy is limited by the maximum tolerated dose to adjacent normal tissues. Thus, effective radiotherapy is considered in terms of how to maximize cancer cell killing capacity within the capacity of acceptable dose that adjacent healthy tissues can tolerate from radiation injury. As cancer is a heterogeneous disease consisting of genetic, architectural, metabolic, pathophysiologic, and immunologic complexities, tremendous efforts have been devoted to identifying biomarkers associated with intrinsic and acquired radioresistance. In the past two decades, many radiation sensitizers and protectors have been identified, allowing radiotherapy to evolve from the traditionally prescribed “one-size-fits-all” concept [4] to a more dynamic and patient-tailored treatment modality.

**Figure 1:**
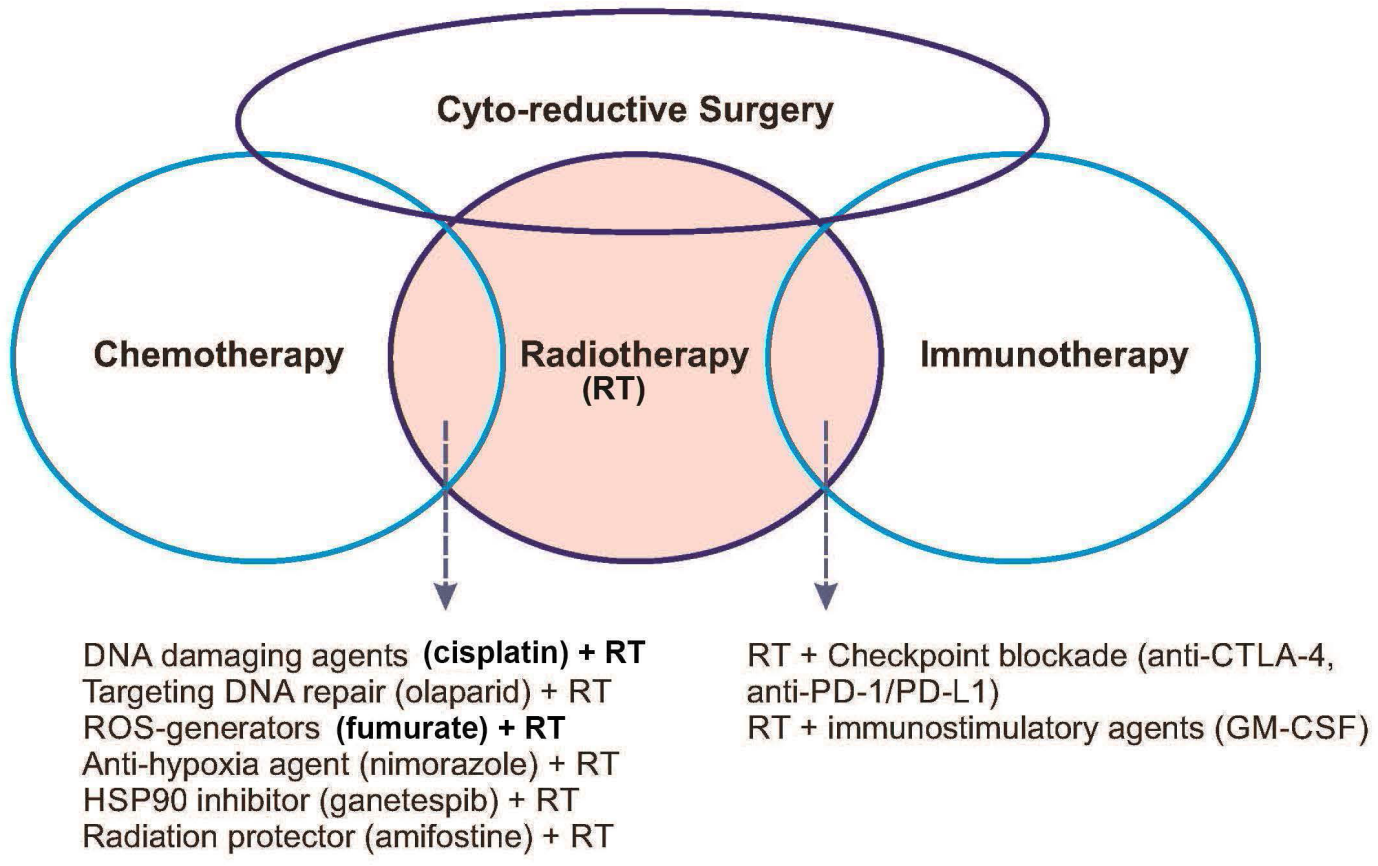
Schematic diagram showing the interrelationships among the four pillars of current cancer therapy, i.e., cyto-reductive surgery, chemotherapy, radiotherapy, and immunotherapy Cyto-reductive surgery is used to debulk tumor mass for subsequent three other treatment types. Radiotherapy is used in combination with many other therapies as indicated in the overlapping areas.

In the era of personalized medicine that patients’ DNA-sequencing and RNA-sequencing data can be precisely determined at the single-cell level. Moreover, new technologies have allowed precisely profiling of protein expression and immune system. These technical advancements enable the realization that tumor cells are highly heterogeneous at the individual patient basis. Using these datasets, radiation oncologists have a large armamentarium with which to develop novel radiation sensitivity markers for improving treatment efficacy. Personalized cancer therapy has been successfully delivered in targeted chemotherapy of chronic myelogenous leukemia with imatinib which targets the BCR-ABL oncoprotein [5]. Other great achievements include the identification of the BRAF mutation as driver in malignant melanoma and the development of vermurafenib which targets BRAF-mutant tumors [6]. Moreover, recent advances in targeted immune checkpoint therapy have firmly established immunotherapy as the fourth pillar in cancer therapy, alongside surgery, chemotherapy and radiotherapy (Figure 1). Radiation can contribute to the alterations of specific and systemic antitumor immune responses, especially for metastatic disease [7]. Therefore, radiation oncology can benefit from cross-fertilizing with other forms of therapy.

In this review, we will describe recent advancements in radiotherapy research in multiple areas, including known radiosensitivity markers such as reactive oxygen species (ROS), DNA repair, tumor microenvironment, as well as newer strategies that integrate cancer genomics/epigenetics and immunology. We keep in mind that preclinical studies are not always clinically sustainable; for example, EGFR inhibitors which have demonstrated synergy with radiation in preclinical studies [8, 9], but failed to so in phase III clinical trials [10, 11]. We will provide an overview of the promises and challenges of current radiotherapy, with a focus on research that is at the stages of clinical validation or has clinical potentials.

## IMPROVING TUMOR ELIMINATION BY TARGETING KNOWN RADIATION SENSITIVITY BIOMARKERS

### Targeting reactive oxygen species

**Figure 2:**
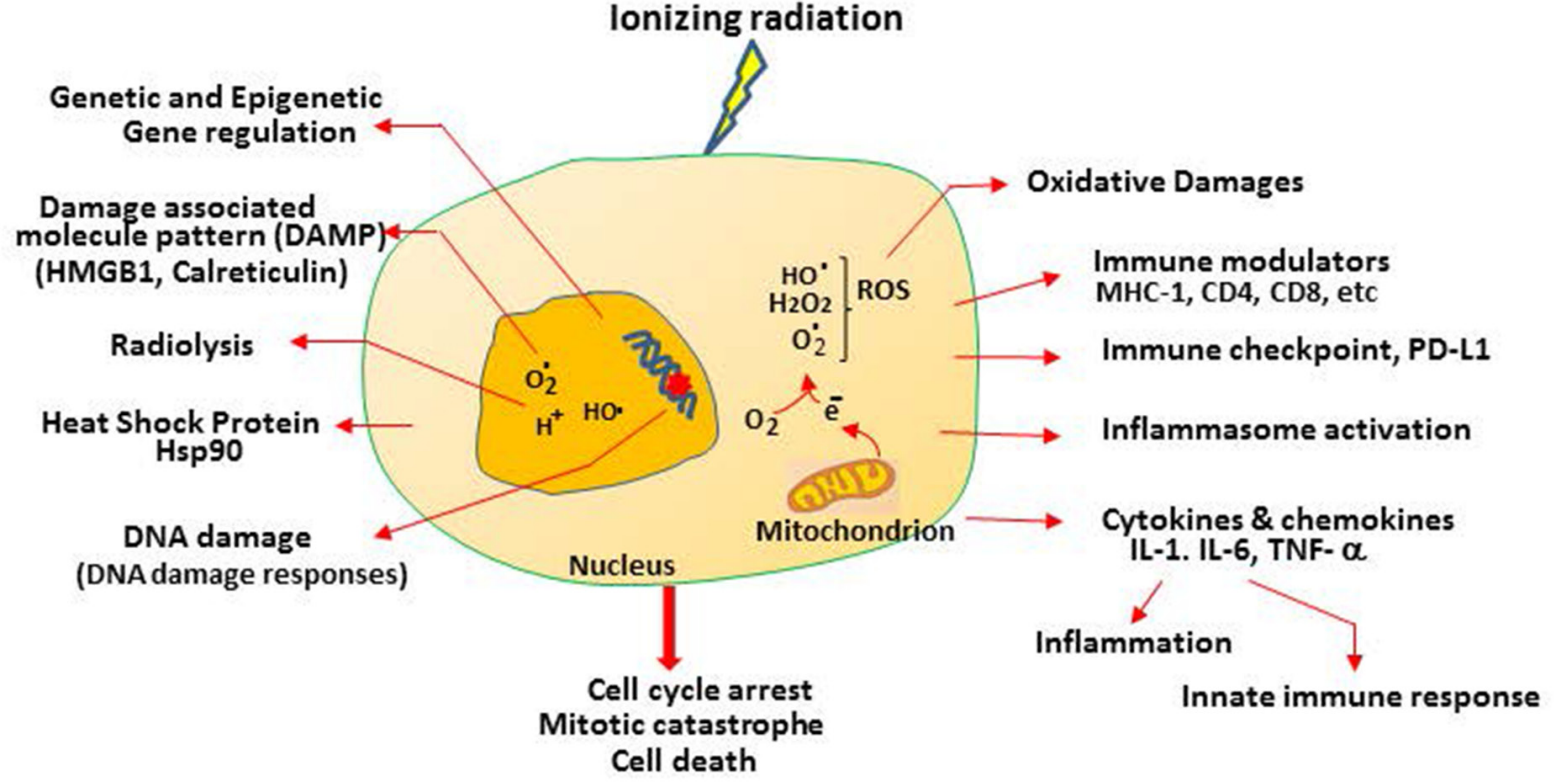
Multiple features of radiation-induced cellular responses in cancer cells Radiation induces radiolysis which “splits” H_2_0 into radicals. This can occur throughout the cells, but for simplicity, only inside the nucleus is indicated. Radiation induces mitochondrial leakage of electrons which interact with O_2_ to generate ROS. ROS can travel into nucleus to cause DNA damage and induce oxidative stress. Other cellular responses include immune modulators, checkpoint, cytokines, inflammation, and DNA damage responses, released tumor antigens and danger signals such as HMGB1 and calreticulin as described in the text.

Ionizing radiation rapidly induces water radiolysis products inside cells, which triggers ROS production [12] (Figure 2). ROS, especially hydroxyl radicals (.OH), play important roles in causing DNA damages and other radiation-induced injury. Mitochondria, which consume about 90% of the body’s oxygen, are the major source of ROS production, mainly from leakage of electrons from the electron transfer chain (complexes I and III, and to a lesser extent complex II) [13]. Radiation-induced ROS production can derive from mitochondria which increases with oxygen tension [14]. Radiation can also induce ROS by activating the cytosolic Rac1/NADPH oxidase system [15, 16]. Cellular redox conditions are regulated by the balance between anti-oxidants and pro-oxidants performed by a host of redox enzymatic reactions. Many preclinical studies have demonstrated that depleting or inhibiting intracellular antioxidants (glutathione, thioredoxin, peroxiredoxin and superoxide dismutase, etc.) can enhance radiation sensitivity; and, in contrast, upregulation of these redox-regulating enzymes can protect radiation damage [17].

Another important pathway involved in protecting radiation-induced oxidative stress is the Kelch-likeECH-associated protein 1-nuclear factor erythroid 2-related factor 2 (KEAP1-NRF2) system. NRF2 is a master regulator of phase II detoxifying and antioxidant genes. Under normal conditions, NRF2 is bound by the adapter protein KEAP1, which recruits the CUL3 ubiquitin ligase, leading to proteasomal degradation of NRF2. During radiation-induced oxidative stress, NRF2 is released from KEAP1, translocates into the nucleus, and transcriptionally upregulates genes involved in anti-ROS functions. Deletion of *Keap1* promotes tumor aggressiveness in animal models and shows resistance to radiotherapy. Moreover, *KEAP1*/*NRF2* mutations are associated with increased risk of local recurrence after radiotherapy in non-small cell lung cancer (NSCLC) patients [18]. Clinical studies have demonstrated correlations between expression of these redox enzymes and poor prognosis of radiation-treated early-stage invasive breast cancer [19].

Several ROS modulators including 2-deoxy-D-glucose and 6-aminonicotinamide [20], curcumin [21] and parthenolid [22] have been demonstrated to enhance relative intracellular redox status. Among these, curcumin has been in several clinical trials as a radiation modulator in treating prostate cancer (NCT1749323) and breast cancer (NCT02724618). Dysregulation of cancer metabolism can also alter cellular ROS status. Germline mutations in the *fumarate hydratase* (*FH*) gene predispose individuals to hereditary leiomyomas and renal cell cancer (HLRCC), which occurs in about 20% of overall renal cell carcinoma (RCC) [23]. The encoded enzyme catalyzes fumarate-to-malate conversion in the TCA cycle. RCC is characterized by fumarate accumulation which promotes the conjugation between fumarate and glutathione, resulting in enhanced ROS production that can be inhibited by the antioxidant, N-acetylcysteine [24, 25]. Cultured cell models have shown that membrane-permeable fumarate (dimethyl fumarate) can enhance radiosensitivity [26, 27]. Although RCC has been conventionally considered radiation resistant, recent clinical studies have shown promising results in primary [28] and metastatic RCC [29]. It would be of interest to investigate whether HLRCC and RCC patients with mutations in FH are particularly sensitive to radiotherapy.

Likewise, free radical scavengers/antioxidants have been developed as radioprotective agents. So far, amifostine (WR2721) is the only radioprotector approved by the US Food and Drug Administration (FDA) specifically in clinical settings to reduce the incidence and severity of acute and chronic xerostomia of patients with head and neck squamous carcinoma without affecting the efficacy of radiation [30]. Amifostine is a sulfhydryl-phosphorylate compound. Upon dephosphorylation by alkaline phosphatase which is preferentially expressed in normal tissues relative to neoplastic counterparts, it is converted into an active metabolite, WR-1065, a free radical scavenger and protector of radiation-induced DNA damages [31]. However, despite its current clinical application, its disadvantages in toxicity and limited route of administration indicate that additional development is needed.

Inflammation is intimately associated with tumor development and progression [32]. Inflammation can promote radiation sensitivity. Inflammasomes, a group of multiprotein complexes consisting of NLRP3, NLRC4, NLRP6, and AIM2, are sensed by a variety of inflammation-inducing stimuli including ROS, to mediate caspase-dependent activation of cytokines [33, 34], leading to a programmed lytic cell death pathway called “pyroptosis”. This is thought the initial host defense mechanism against infection by exposing intracellular pathogens to the innate immune system [35]. Inflammasomes (Nlrp3, caspase 1 et al) are significantly induced in the lung of mice by irradiation, and induced inflammasomes accelerate radiation-induced inflammation and pneumonitis and fibrosis in animal model [36]. *Nlrp3*^*-/-*^ cells are resistant to radiation-induced DNA damage [37], and so are mice lacking caspase 1. ROS-induced DNA damage can be sensed by the AIM2 inflammasome, which senses double-stranded DNA breaks [38, 39]. *Aim2*^*-/-*^ mice exhibit intestinal protection from lethal effects by subtotal body irradiation [40]. These findings suggest that targeting these inflammasomes may be an effective strategy for cytoprotection against radiation-induced lethality.

### Targeting radiation-induced DNA damage response signaling

Radiation-induced DNA damage of normal tissues, if not properly repaired, contributes to the major mechanism of cell death [41]. Mechanisms of radiation-induced DNA damage and repair have been extensively investigated over the past two decades [42, 43]. Radiation-induced DNA damage includes abasic lesions, deoxyribose ring opening, single-stranded breaks and double-stranded breaks (DSB). Different sensing mechanisms are involved in different types of DNA damage responses (DDRs). Ataxia telangiectasia mutated (ATM), together with MRE11-RAD50 and the NBS1 complex are the earliest responders to DSB. Cells and animal models have demonstrated that defective breast cancer gene 1/2 (*BRCA1/2)* which encodes the repair system for DNA DSB is involved in radiation resistance. Clinical trials showed that breast cancers with *BRCA1 (-/-)* exhibit elevated sensitivity to radiation therapy [44].

Single-stranded breaks are recognized by poly(ADP-ribose) polymerase 1 (PARP1) for the synthesis of a poly-(ADP-ribose) chain for recruiting the factors X-ray cross-complementing protein 1 (XRCC1) and DNA polymerase β (Polβ) for DNA repair. Several targeting PARP agents are at various stages of clinical development [43]. Olaparib, an anti-PARP1 agent, has been approved by the FDA for treating ovarian and breast cancers with *BRCA* mutations, often in combination with platinum-based chemotherapy [45]. Olaparib resistance due to upregulation of multidrug resistance drug efflux mechanism which eliminate olaparib and also due to secondary mutation of BRCA2, are frequently associated with olaparib failure [43]. It has been tested in clinical trials in combination with radiotherapy against NSCLC, breast cancer, and head and neck cancer. Another PARP inhibitor rucaparib was approved by the US FDA for treating ovarian cancers in December, 2016 [46].

Pitroda et al reported that reduced expression of a four-gene signature involved in DNA repair pathway (*Rif*1, *PARI*, *RAD51*, and *Ku80*) is associated with reduced patient survival rates and adverse clinical features in breast cancer and NSCLC [47]. Heat-shock protein 90 (Hsp90), which is involved in correcting protein misfolding of its client proteins, directly regulates about 725 client proteins, many of them are related to DDR signaling, e.g., ATM, NBS1, and ATR [48]. In addition, numerous publications have indicated that inhibition of Hsp90 results in radiation sensitization in cultured cell models ([49, 50] and references therein). The Hsp90 inhibitor, ganetespib, has been in clinical trials as a radiosensitizer in rectal cancer (NCT01554969) and esophageal cancer (NCT02389751). Ganetespib is safe and well-tolerated, lacking cardiac, liver and ocular toxicity. It has the potential to become the first-in-kind Hsp90 inhibitor to be approved as a radiosensitizer [51]. On the whole, after many years of research, it appears that targeting DDR signaling remains a viable approach for improving radiation sensitivity.

### Targeting tumor hypoxia

Hypoxia is a hallmark of solid tumors due to their poor vascularity, and is associated with poor prognosis in many types of cancers, including cervical carcinoma [52], head and neck cancer [53], and sarcomas [54]. Hypoxia is associated with increased chemoresistance, genomic instability, and tumor invasion and metastasis [55]. It was estimated that hypoxic cells can be up to three times more resistant to radiation-induced damage than aerobic cells are [53, 56]. Hypoxic tumors can be detected using the FDA approved fluorine-18 fluoromisonidazole by positron emission tomography. Hypoxic radiosensitizers such as hyperbaric oxygen, carbogene breathing and nitroimidazoles [57] have been tested in clinics to sensitize radiation and have been associated with improved locoregional control and disease-free survival compared with radiation alone for head and neck squamous cell carcinoma (HNSCC) [58].

Nimorazole, a radiosensitizer by fixation of radiation-induced damage under hypoxic conditions [59], has showed improved the 5-year actuarial rate of loco-regional control of HNSCC from 33% (placebo) to 49% [60] in a randomized Danish trial. It has thus been adopted for routine clinical use in head and neck cancer patients in Denmark. However, results from other phased III trials were not as impressive [61]. The use of DNA-damaging agent cisplatin in a nimorazole and radiation combination trial showed additional improvement of HNSCC to 80% [62]. Tirapazamine (TPZ) has been shown to preferentially kill hypoxic cells because of its activation under low O_2_ conditions [63]. TPZ has been shown to enhance cisplatin-induced cell killing in a mouse tumor model, however, a phase III trial (TPZ, cisplatin, and radiation versus cisplatin and radiation) for HNSCC showed no evidence of improved overall survival with TPZ [64]. Thus, despite many years of exploratory studies, it appears that targeting hypoxia for radiation sensitization using hypoxia modifiers has yet to produce clinical impact and further investigation remains needed.

## IMPROVING RADIOTHERAPY BY EXPLOITING NEW TARGETS IN CANCER GENOMICS

### Identifying a gene signature for radioresponse prediction

Recent advances in genomic analyses in patients have brought precision oncology close to reality. This development has been primarily due to the affordable next-generation sequencing (NGS) and advancement in bioinformatics. NGS has been routinely incorporated into clinical care. For example, in a recent study of 213 patients with HNSCC who have been followed routine care including surgery, definitive radiation, or definitive chemoradiation. It was found that PIK3CA amplification (but not mutations) and RAS mutations were associated with poorer outcomes [65]. In another study, molecular profiling of 151 advanced and treatment-resistant HNSCC cases using an NGS platform identified actionable targets that may guide treatment decision-making [66]. Recent large-scale genome-wide association studies and large-scale replication genotyping identified more than 90 breast cancer susceptibility loci [67, 68]. Patients with genetic association with high polygenic risk of breast cancer have no increased risk of developing late or acute radiotherapy toxicity [68].

In another radiotherapy study, 58 prostate cancer patients without prior treatment received IMRT with a targeted dose of 61 to 72 Gy. This study identified 19 genes with elevated steady-state mRNA levels that were correlated with radiation-resistance. Most of these genes are in the DSB repair pathway [69]. Eschrich et al developed a radiation sensitivity molecular signature based on gene expression profiling datasets and identified 10 genes out of an original pool of more than 7000 genes. This signature has been validated in 5 independent cohorts of 621 breast cancer patients [70]. Similarly, Zhao et al [71] developed a 24-gene post-operative radiotherapy outcomes score (PORTOS) for monitoring the likelihood of developing postoperative radiotherapy metastasis at 10 years in patients with prostate cancers. It was found that patients with high PORTOS were less likely to have metastasis at 10 years than those who did not receive radiotherapy.

In addition to transcriptional regulation, there is evidence that epigenetic mechanisms are also involved in radiosensitivity. Ahmed et al. reported that elevated expression of O(6)-methylguanine-DNA methyltransferase (MGMT) is significantly associated with radiosensitivity in a subset of glioblastomas [72]. In a prostate cancer cell model, it was reported that irradiation caused durable upregulation of cancer stem cell marker proteins, including aldehyde dehydrogenase 1A1 (ALDH1A1) as well as long-term altered histone methylation patterns of H3K4, H3K36, and H3K27 tri-methylation [73]. Alterations in epigenetic marks play a substantial role in radiation response and in fibrosis development (see below) [74].

### Identifying radiation toxicity genes in normal tissues

Radiation toxicities in normal tissues include acute radiotoxicity and late complications such as telangiectasia, edema, and fibrosis. Of particular importance is radiation-induced fibrosis, a late event usually occurs 4 months to several years after radiation. Fibrosis can occur in many organs depending on dose, volume of irradiated tissue, and types of tissue exposed to irradiation. As the cancer patients has increased survival time, this can significantly deteriorate quality of life or even causes death [74]. Radiation-induced fibrosis has been proposed to follow a mechanism similar to that of chronic wound healing processes [75, 76]. Radiation-induced fibrosis is considered to arise from complex molecular signaling involving cytokines, growth factors, integrins and cell adhesion, stress response and DDR, and extracellular matrix remodeling, resulting in the formation of altered cell architecture called myofibroblasts [74].

Several studies have described differences in gene expression profiles between samples derived from patients with and without radiation-induced fibrosis using patients-derived fibroblasts in cultures [77, 78] or peripheral lymphocytes [79, 80]. However, these studies have not generated confirmatory “gene expression signatures” for predicting radiation-induced fibroblasts. Recently, in a gene expression profiling of whole blood from breast cancer survivors with and without fibrosis 3-7 years after radiation therapy was published and 87 differentially expressed genes were identified, including genes downregulated during the maintenance phase of fibrosis as opposed to genes upregulated during the early, initiating phase of fibrosis. Genes involved in the TGF-β1 signaling were frequently upregulated [81].

Several preclinical approaches have been designed to target radiation-induced fibrosis in various organ sites in mice, either by targeting matrix synthesis or by suppressing inflammation [76]. In one lung cancer model, it was found that TGF-β1 receptor inhibitor LY2109761 showed reduction of radiation-induced inflammation and pulmonary fibrosis and prolonged survival [82]. These findings led to a phase I/II trial using stereotactic ablative radiotherapy in combination with anti-TGF-β1 antibody (fresolimumab) in SCLC patients to investigate whether fresolimumab inhibits radiation-induced cytotoxicity (NCT02581787). In a randomized clinical trial in which pentoxifylline and vitamin E were given for 6 months after breast wall irradiation, no difference in overall survival and disease-free survival was observed, however, fibrosis was significantly reduced in the treated group [83].

## RADIOTHERAPY, TUMOR RECURRENCE, AND CIRCULATING TUMOR CELLS

Circulating tumor cells (CTCs) are rare cancer cells shed from primary or metastatic sites. One mechanism of cancer cells release into the circulation may be due to radiation-induced structural damage to blood vessels within the tumors. CTCs have provided non-invasive “liquid biopsies” for cancer molecular and immune diagnoses, and also real-time monitoring of treatment response that may guide the treatment options [84]. Several pre-clinical studies have shown that radiotherapy can enhance CTC production in animal models, and CTC density is an independent biomarker for poor prognosis in NSCLC [85], esophageal squamous cell carcinoma [86], and breast cancers [87]. This may be particularly relevant in the early stages of hypofractionated radiotherapy when accumulated radiation dose is not sufficient to kill most cancer cells, suggesting that hyperfractionated radiotherapy may be more effective in eliminating CTCs. Indeed, in a randomized controlled trial involving 563 NSCLC patients that were treated with a high-intense but brief regimen called continuous hyperfractionated accelerated radiotherapy (CHART) vs conventional prolonged radiotherapy. CHART regimen showed significant improvement in achieving local tumor control and survival than the conventional hypofractionated radiotherapy [88]. However, in another study, hyperfractionated radiotherapy showed no superiority to conventional radiotherapy in childhood medulloblastoma. These results suggest that radiotherapy efficacy may depend on fractionated schemes including tumor types and radiation schedules [89].

It has been reported that CTCs in the blood can re-colonize back at the original site, where cytokines are produced which serve as CTC attractants [90]. This “self-seeding” has been documented in breast cancer after irradiation, resulting in tumor recurrence after radiotherapy. Granulocyte-macrophage colony stimulating factor induced upon irradiation is one cytokine that stimulates tumor self-seeding [91]. It has been reported that about half of the breast cancer patients after conserving surgery and radiotherapy, have recurrence disease at the same site [92]. Although this tumor recurrence cannot be entirely due to CTCs, these observations suggest that CTCs play dual roles in tumor dissemination and tumor recurrence after radiation.

Advances in DNA-sequencing and RNA-sequencing technologies at the single-cell level have made CTCs important cell sources for genomic landscape profilings. Although the utility of liquid biopsy analysis for biomarker identifications in radiotherapy sensitivity/resistance and radiation toxicity remains to be established, it is conceivable that CTCs will play an important role in this area.

## IMPROVING RADIATION SENSITIVITY THROUGH MODULATION OF TUMOR IMMUNITY

### Radiation and general immune response

Radiotherapy was previously considered immunosuppressive because older treatment techniques covered large fields that caused substantial damage to bone marrow and circulating blood [93]. However, it has become clear that radiation can elicit multiple forms of host immune responses affecting the efficacy of radiotherapy [94–96]. Radiotherapy in sarcoma patients induces upregulation of several positive immune effectors, such as NKG2D, CD45, CD3, CD4, CD8, MHC-II, β2M, perforin, granzyme B, the CT-antigen CT7 and macrophages (CD68, CD163) in a coordinated fashion following radiotherapy, whereas transcripts associated with immune suppression such as IDO, BTLA, FoxP3, PD-L1, IL-10, TGF-β, STAT-3, CT10, TNF-α, and iNOS seem to follow a similar pattern of downregulated expression after radiotherapy [97].

**Figure 3:**
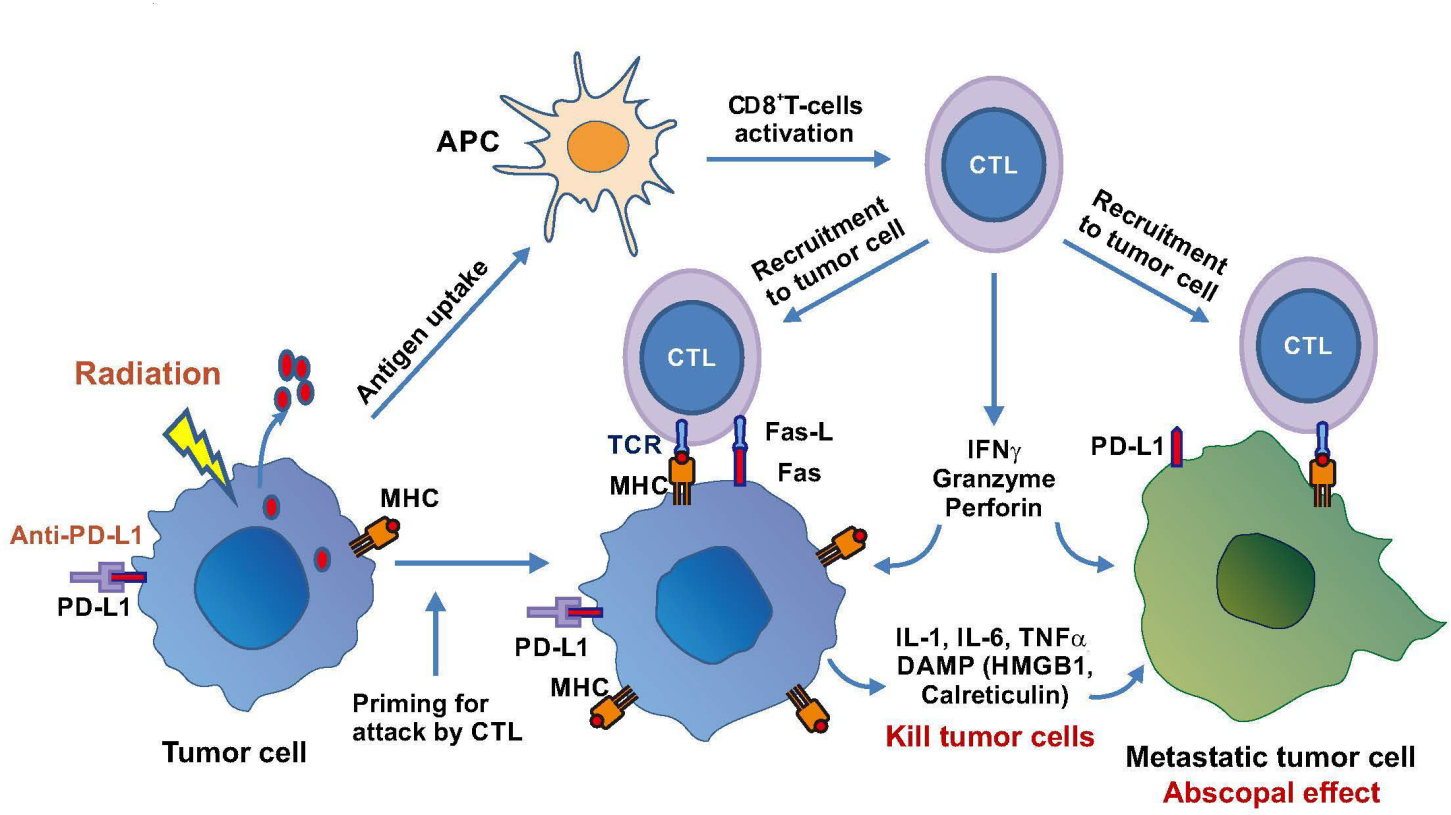
Radiation-induced immune response in cancer therapy Radiation induces release of tumor antigens which are captured and processed by antigen-presenting cells (APC) to activate cytotoxic T lymphocytes (CTL). CTL are recruited to attack tumor cells or metastatic cells to elicit abscopal effect. Radiation also upregulates PD-L1 and combination with anti-PD-L1 therapy enhances MHC and Fas expression on tumor cells and increases T-cell-mediated cytotoxicity including releases of cytokines and DAMP to elicit killing of primary and metastatic tumor cells.

Among these, the immune effector, MHC-I has been the best studied [98–100]. MHC-I presents peptides of 10 to 12 amino acids from a variety of cellular proteins after immunoproteasomal degradation onto the cellular surface via antigen-presenting cells (APC) for the recognition of and activation of CD8^+^ cytotoxic T cells (Figure 3). MHC-I is normally downregulated in many types of solid tumors for immune-surveillance by the host [101], and MHC-1 downregulation often leads to activation of NK cell killing of target cells [102]. Mechanisms of MHC-I silencing in tumor cells are multifactorial, but one important mechanism is epigenetically regulated including DNA methylation [103, 104] and histone acetylation [105]. β2 microglobulin encodes a component of the MHC-I complex. Increasing MHC-I expression by gene delivery of β2 microglobulin has been a strategy for increased tumor immunogenicity [106]. γ-radiation induces MHC-I expression, increase of intracellular peptide pool, antigen presentation, and cytotoxic T lymphocyte recognition of the irradiated cells [98].

Cytotoxic T cells, when activated, produce immunotoxins such as perforin (a pore-forming cytolytic protein allowing passage of proapoptotic proteases), granzymes (a family of serine proteases which activate apoptosis by caspases) and granulin (which is present in cytolytic granules of CTL and NK cells). These cytotoxins enter the cytoplasm of the target cells and elicit cell death mechanisms. Activated T cells are able to home-in and infiltrate the tumor; inefficient T cell migration is a major barrier of cancer immunotherapy. Radiation has been shown to promote these processes [107]. In a B16 murine melanoma model, it was demonstrated that increased production of infiltrating CD45^+^, CD8^+^ T, and tumor-specific CD8^+^ T cells, and depleting CD8^+^ T cells completely abolished the therapeutic effects [95]. In a preclinical pancreatic tumor model, in which infiltrated T cells are low, local low-dose irradiation induces recruitment of tumor-specific T cells through direct radiation effects on the tumor tissues [108]. Another important interaction between radiation and immune system is upregulation of the cell surface death receptor Fas by sublethal irradiation [109]. Fas, by interacting with its ligand FAS-L, induces caspase activation-mediated apoptotic cell death [110]. These findings demonstrate the importance of radiation therapy in cancer immunotherapy and suggest that responses to immunotherapy may be improved by harnessing radiation (Figure 2).

It has been well-established that locoregional irradiation can generate both local and distant effects at sites away from irradiation or the “abscopal effects” [111–114]. The abscopal effects have been attributed to the induced inflammatory cytokines such as IL-1β, IL-6, and TNF-α released from the primary tumor site or non-tumor cells such as endothelial cells and cancer associated fibroblasts (Figure 3) [115]. These cytokines elicit cellular stress and induce damage-associated molecular pattern (DAMP) molecules which alert the host of danger by triggering the defense system to protect cells from killing effects. High mobility group box 1 (HMGB1), which binds to toll-like receptor on dendritic cells and promotes antigen cross-presentation onto the surface of the T cells, is a well-known DAMP molecule [116] (Figure 1). Another DAMP molecule is calreticulin whose main function is to bind misfolded proteins, preventing them from exposing to the endoplasmic reticulum or Golgi apparatus. Enhanced release of HMGB1 and increased calreticulin expression are important for priming antigen-specific T-cell responses, thereby promoting synergistic effect between radiation and immunotherapy (Figures 2 and 3). Damaged cells induced by irradiation may also increase production of death receptor Fas for killing by tumor antigen-reactive T cells as mentioned above.

### Combined radiotherapy and immunotherapy

The development of antibody blockade of immune checkpoint regulators such as cytotoxic T-lymphocyte-associated protein 4 (CTLA-4) and the program death-1 and its ligand (PD-1/PD-L1) axis has revolutionized cancer immunotherapy [117]. CTLA-4 is homologous to CD28. The CTLA-4 receptor is located on the surface of effector T lymphocytes and interacts with CD80/CD86 (B7-1 or B7-2) on CTLA-4 antigen-presenting cells to induce T cell rest, preventing overactivation of the T cell system in autoimmunity [118]. CTLA-4 is up-regulated in response to T-cell activation and induces an inhibitory signal in the T cells. Ipilimumab, a monoclonal antibody against CTLA-4, was approved for treating human melanoma in 2011. Likewise, PD-1, another cell surface receptor, also acts as an inhibitory immune checkpoint. It is expressed on CD8^+^ and CD4^+^ T cells and antigen-presenting cells. The ligands of PD-1 are PD-L1 and PD-L2, and anti-bodies against these ligands (atezolizumab and durvalumab) have been developed. When bound to its ligands PD-L1/PD-L2, they elicit a T-cell inhibitory signal through inhibition of T cells costimulastory receptor CD28 but not T cell receptor (TCR) as previously believed [119, 120]. Humanized monoclonal antibodies (penbrolizumab and nivolumab) have been approved for clinical use. Other potential immunoregulatory receptors for immunotherapy under developed are OX40 (CD134) and TIM3 [121].

Preclinical studies have demonstrated that anti-PD-1/PD-Li plus radiation induces cytotoxic T lymphocyte activation but suppression of myeloid-derived suppressor cells in a mouse model [122, 123]. In a murine lung cancer model resistant to PD-1 antibody established by repetitive dosing shows increased expression of the antigen presentation pathway, including MHC-I and MHC-II, and reduced CD8^+^, CD4^+^ lymphocytes and infiltrating lymphocytes. Local tumor radiation in this model induces IFNβ and MHC-I production and resensitization to anti-PD-1 antibody therapy. These results suggest that radiotherapy can overcome PD-1 resistance via upregulation of the MHC system [124]. These preclinical results showed that radiation and immune checkpoint blockade possess promise for synergistic effects.

A synergistic effect of immunotherapy combined with radiotherapy is the abscopal effect. This has been reported in melanoma patients and lung patients using radiotherapy in combination with ipilimumab [113]. Lung cancer patients treated with radiotherapy and ipilimumab increases tumor-infiltrating cytotoxic lymphocytes which cause tumor regression [125]. In a phase I trial using stereotactic ablative radiation therapy with ipilimumab involving 32 patients, it was demonstrated that clinical benefit was associated with increased peripheral CD8+ T-cells, CD8+/CD4+ T-cell ratio, and proportion of CD8^+^ T-cells expressing 4-1BB and PD1 [126]. In another study using local radiotherapy combined with systemic ipilimumab immunotherapy in 22 patients with stage IV melanoma showed that 50% of the treated patients benefited from the treatment, and response was correlated with elevated CD8-activated T-cells, suggesting abscopal effects from the treatment. Further improvement in the treatment effects was observed in radiation plus dual immune checkpoint blockade (anti-CTLA-4 and anti-PD-L1), suggesting non-redundant mechanisms in this triple treatment scheme. It was revealed that CTLA-4 blockade primarily decreases Treg cells, PD-L1 blockade predominantly reinvigorates exhausted CD8^+^ TILs, and radiation diversifies the TCR repertoire of TILs from unirradiated tumors [127].

Preclinical studies have demonstrated that dose and schedule of radiotherapy are important in eliciting anti-tumor activities, and this is the case with immune checkpoint inhibition as well. In general, hypofractionated radiotherapy has been found to be more effective than single high-dose radiotherapy in generating anti-tumor immune response with anti-CTLA-4 [128]. Fractionated radiotherapy in combination with ipilimumab have been used in clinical studies [129–131]. Results from these studies suggested that subsets of patients have more favorable responses. These findings encourage further investigations using radiation and immunotherapy in cancer treatment.

Currently, there are about 20 active clinical trials combining radiation with CTLA-4 inhibition and more than 50 trials combining radiation with PD-1/PD-L1 therapy in a wide range of malignancies [96]. Metastatic melanoma is the most common indication in these trials. While the results of these ongoing trials are not yet available, this enthusiasm should generate ample information for designing optimal dosing and sequencing of radiation treatments. The results may also provide new biomarkers for optimizing treatment sensitivity while minimizing overlapping toxic effects of radiation and immunotherapy.

### Personalized radiation-immunotherapy, is it feasible?

An important hallmark of cancer is genomic instability, which is the fundamental mechanism for the generation of neoantigens. Neoantigens are processed and presented on the surface of neoplastic cells by the pathways of MHC systems and recognized by CD8^+^ TCR. It has been demonstrated that patients with genomic instability traits fare better with immunotherapy using PD-1 [132] and CTLA-4 blockade [133, 134]. Consistent with these findings, a recent report showed that melanoma patients with high mutation loads as determined by NGS responded better to anti-PD1/PD-L1 therapy [135]. Thus, neoantigen load may be considered a biomarker in cancer immunotherapy [117, 136]. Radiation damage would produce large amount of tumor neoantigens for presentation by antigen-presenting cells. Moreover, such damage would activate inflammatory signals to induce antigen-presenting maturation and migration to activate naïve T cells [137].

Neoantigens raised from non-synonymous mutations of tumor DNA [117, 138], generating mutated peptides that are presented by the MHC system to induce CD4 and CD8 T cells responses. Recent studies have demonstrated correlations between abundance of tumor neoantigens (or neoantigen load) and microsatellite-instability status in colorectal cancers [139] and endometrial cancer [140]. Strikingly, a stronger association between clinical response and mismatch repair deficiency than that with mismatch repair proficient tumors to PD-1 blockade was found in advanced urothelial carcinoma [141]. Additionally, melanoma bearing high-mutation loads showed improved overall survival with anti-PD1, and tumors from responding patients are enriched in mutations in the DNA repair gene BRCA2 [142].

The size of the neoantigen repertoire with T cell reactivity is not known. However, with the advancement of deep-sequencing and mass spectrometry technology, more and more patient-specific neoantigens are beginning to unravel. Recently, Riaz et al have found two neoantigens, SERPIN3 and SERPINB4, the human ovalbumin antigen, which are associated with autoimmunity, are also correlated with improved survival after anti-CTLA-4 immunotherapy in patients with melanoma [143].

Neoantigens derived from tumor-specific lymphocytes have been identified in the circulating bloods of melanoma patients. It was previously demonstrated that tumor-infiltrating T cells in human melanoma are enriched in PD-1^+^ subpopulation [144]. Because the scarcity of the cell source, the authors first isolated a CD8^+^ PD-1^+^ population from patients using flow cytometry. Neoantigens were determined by deep-DNA sequencing of patients’ tumor specimens. These observations have important implications for providing a noninvasive and simplified strategy for producing of patients specific TIL for immunotherapy.

These observations suggest that exposures to mutagens such as ultraviolet light in melanoma and tobacco smoking [145] in NSCLC may increase mutational loads leading to increased immunogenic neoantigens production. Indeed, these have been found in clinical data [146–148]. In a recent study involving whole exome sequencing of 16 pairs of matched specimens of squamous cell carcinoma of anal canal cancer from patients before and after recurrent disease treated with concurrent chemotherapy and radiotherapy, while overall mutational spectra were not significantly different between pre- and post-treatment tumors, one patient with recurrent disease was exceptional response to anti-PD-1 therapy. High mutational burden and predicted neoantigen load were observed in the tumors [149]. More studies are needed to better understand mutational dynamics and neoantigen presentation in response to radiation-immunotherapy. These studies may lead to successful personalized radiotherapy in the future.

## CONCLUSIONS AND FUTURE PERSPECTIVES

Two major driving forces have come together that improved the treatment efficacy of radiotherapy in recent years. One is the advancement of technology of dose conformity such as IMRT, SBRT, and IGRT, allowing more precise delivery of high-dose radiation to the target volume with reduced injury on healthy tissues. And the other is a better understanding of radiosensitivity/resistance mechanisms at the molecular and cellular levels which enable the development of radiosensitizers and radioprotectors. Radiotherapy was traditionally used in combination with chemotherapy or surgery in treating human malignancies. Taking advantage of successful checkpoint blockade immunotherapy in multiple tumor types, radiation-immunotherapy has become an area of intensive ongoing investigations and the overall results will soon be available.

As we move into the era of personalized medicine, the future of radiotherapy decision-making will continue to evolve by integrating information based on individual patient’s DNA data, gene and proteomic expression profilings, tumor metabolomes, and immunology. Combining traditional mechanism-based radiation oncology with new knowledge derived from these new developments will continue to improve the treatment efficacy using multiple treatment modalities and radiotherapy will continue to play an important part in these modalities.

While locoregional tumors can be effectively treated by radiotherapy, metastatic cancers are difficult to treat and representing an important challenge in radiotherapy. Effective abscopal response in radiotherapy is critical to combating widespread metastatic disease. While therapeutic efficacy has often emphasized the overall survival and progression-free rates, radiation-induced toxicity in the normal tissues cannot be neglected. Radiation can induce adverse effects such as fibrosis in normal tissues and immune toxicity such as exacerbation of autoimmunity and inflammation, resulting in pulmonary pneumonitis when combined with immunotherapy. Both can affect quality of life and may even lead to death. Thus, the traditional concept of a “therapeutic window”, which refers to the dose range that provides maximal effective therapy without harmfully affect normal tissue, is of great importance. Like many other forms of cancer therapy, radiotherapy must constantly strive to widen the therapeutic window to improve overall treatment efficacy. Uncovering and effectively utilizing new knowledge from multiple disciplines to optimize radiotherapy is critical to this endeavor. Although much work remains to be done, new information continues to pour-in that can benefit radiation research. The future for radiation therapy looks bright.
